# A real-life evaluation of SNOT-22 domains in a cohort of CRSwNP patients treated with biologic therapies for 12 months

**DOI:** 10.1016/j.waojou.2025.101041

**Published:** 2025-03-11

**Authors:** Giulia Anna Maria Luigia Costanzo, Andrea Giovanni Ledda, Giada Sambugaro, Giuseppe Murdaca, Cristiano Caruso, Silvia Canalis, Paolo Serra, Maria Pina Barca, Stefano Del Giacco, Davide Firinu

**Affiliations:** a Department of Medical Sciences and Public Health, University of Cagliari, Azienda Ospedaliero Universitaria, SS 554-Bivio Sestu, 09042, Monserrato, CA, Italy; b Department of Internal Medicine, University of Genoa, Italy; c Allergology and Clinical Immunology Unit, San Bartolomeo Hospital Sarzana, Italy; d UOSD DH Medicina Interna e Malattie dell'Apparato Digerente, Fondazione Policlinico A. Gemelli IRCCS, 20123 Rome, Italy; e Department of Medical Sciences and Public Health, Faculty of Medicine, Università Cattolica del Sacro Cuore, 00168 Rome, Italy

**Keywords:** CRSwNP, SNOT-22, Asthma, Biologicals, Monoclonal antibodies

## Abstract

**Background:**

Chronic rhinosinusitis with nasal polyps (CRSwNP) is an inflammatory disorder associated with rhinorrhea, nasal obstruction, nasal congestion, hyposmia, anosmia, and facial pain or pressure for over 12 weeks. This study examines the Sino-Nasal Outcome Test 22 (SNOT-22) score and its relationship to nasal, otologyc, sleep and emotional domains in CRSwNP patients during the first year of biologics treatment, comparing the pre-biologics score to that at 1, 6, and 12 months in a cohort of 59 patients with CRSwNP.

**Methods:**

We included 59 patients with CRSwNP (with or without asthma) who received add on therapy with targeted monoclonal antibodies (mAbs). At each visit we administered the SNOT-22 questionnaire and both total score and single domains scores were recorded.

**Results:**

In this real-life, observational study, we found a significant SNOT-22 total score reduction for patients treated with anti-IgE after 1 month, but this significant difference was not maintained at 6 or 12 months compared with the baseline. The use of anti-interleukin 5/5R (IL5/5R) leads to a significant reduction of the SNOT-22 total score after 1 month, which is maintained after 6 months but not at 12 months compared with the baseline. The use of an anti-interleukin 13/4R (IL13/4R) leads to a statistically significant reduction of the SNOT-22 score after 1 month of therapy, which is maintained after 6 and 12 months compared with the baseline. When examining the single domains, we observed that patients who received anti-IL13/4R treatment demonstrated a significant reduction in each domain at each time point (T) compared to the baseline. Patients who received anti-IL5/5R treatment demonstrated an improvement in the nasal domain at each T compared to the baseline. However, the improvement in the otologyc domain was not sustained after 12 months. Similarly, the sleep domain remained unchanged, and the emotional domain only improved significantly after 12 months. Similarly, there was a reduction of the emotional domain in patients treated with anti-IgE.

**Conclusion:**

Our real-life study describes the kinetics over the first year of treatment with mAbs in CRSwNP, showing different patterns in reducing symptoms and improving Health Related Quality of Life (HRQoL). SNOT-22 with the factorial division in 4 domains can help distinguish fast responders from low or non-responders to a mAb based on clinical response after 1 month and more accurately assign the right mAb to the right patient.

## Introduction

Chronic Rhinosinusitis with Nasal Polyps (CRSwNP) is an inflammatory disease defined by severe symptoms such as rhinorrhea, nasal obstruction, nasal congestion, hyposmia and anosmia, and facial pain or pressure for a duration of more than 12 weeks.^1^ CRSwNP is a significant phenotype of Chronic Rhinosinusitis (CRS), having a 2.1–4.3% frequency in Europe and 1% in China that variably encloses asthma, allergy or Non-steroidal anti-Inflammatory drugs-Exacerbated Respiratory Disease (N-ERD). Until recent years, the available therapies such as intranasal corticosteroids (INCS), systemic corticosteroids (SCS), and surgery frequently failed to adequately manage symptoms, resulting in a considerable loss in health-related quality of life (HRQoL).^2^ The high frequency of relapses or exacerbations after surgery, the frequent comorbidity with late-onset asthma, reduce significantly the HRQoL. In patients with severe CRSwNP this reduction is of similar importance of that reported in other chronic diseases such as rheumatoid arthritis or diabetes.^3^ The clinical, economic, and psychological impact of this condition and their behavior after treatments are still partially understood.^4^

In Western countries, CRSwNP is mostly associated with a type 2 inflammation pattern, involving the interleukins IL-4, IL-5, and IL-13, as well as eosinophil, basophil, and mast cell infiltration of mucosa and nasal polyps.^5^ Clinical epidemiological and pharmacological studies suggested that CRSwNP and asthma are inextricably linked and frequently coexist.^6^^,^^7^ Inflammation in the nasal mucosa and lower airways are strongly linked,^8^ and a correlation between the inflammatory profile of nasal and bronchial biopsies in patients with CRSwNP has been observed.^9^ Other evidence of a link between CRS and an impaired HRQoL has been found in patients with Severe Asthma (SA), where there is a significant positive correlation between sinusitis severity and lower airways inflammation.^10^

Patients with CRSwNP with concurrent asthma have a more severe illness, presenting higher rates of nasal polyp recurrence and corticosteroid dependence compared to patients with isolated asthma. Asthma is also harder to manage when CRSwNP is present due to more severe obstruction and eosinophilic inflammation.^11^

Biologic therapies that target type 2 inflammation, represented a paradigm shift in the therapeutic approach for patients affected by severe and refractory CRSwNP.^12^ Their use is indicated in patients with bilateral polyps that previously underwent to Endoscopic Sinus Surgery (ESS) or that do not fit the criteria for surgery, having 3 criteria between: evidence of type 2 inflammation, need for (or contraindication to) administration of SCS, a significantly impaired HRQoL, a significantly impaired sense of smell, and/or comorbid asthma.^13^

The anti-IgE monoclonal antibody, omalizumab, was the first biologic therapy authorized in Italy for SA. It has been shown that it reduced the rate of exacerbations and the number of hospitalizations, and it improved the Asthma Control Test (ACT) score.^14^ Patients treated with omalizumab, also, displayed reductions in CRS symptoms, endoscopic Nasal Polyp Score (NPS), and as-needed INCS in randomized and real-life studies.^15^, ^16^, ^17^ In the pooled analysis of POLYP 1 and POLYP 2 trials the Sino-Nasal Outcome Test 22 (SNOT-22) score improved of 23.1-point in patients treated with omalizumab compared to 7.7 in those in the placebo arm.^18^ Currently, in Italy, omalizumab is authorized also for CRSwNP.

Mepolizumab is a humanized mAb against interleukin 5 (IL-5), which reduces the number of eosinophils in the sputum and peripheral blood.^19^ The MENSA study found that treating patients with mepolizumab reduced asthma exacerbations by 50% improved HRQoL and resulted in better asthma control in patients with Severe Eosinophilic Asthma (SEA).^20^ Furthermore, according to the SYNAPSE trial, mepolizumab is safe and effective as add-on to standard of care of refractory, severe, and bilateral CRSwNP candidates for surgery. Patients who were given mepolizumab had a significant decrease of SNOT-22 score, lowering it by about twice the Minimal Clinically Important Difference (MCID) for SNOT-22 (8.9).^21^ The effects were detectable 4 months after starting therapy and lasted until week 52.^22^ Mepolizumab is currently approved in Italy for both SEA and severe CRSwNP. Benralizumab is a mAb that targets the IL-5 receptor (IL-5R) on eosinophils, eosinophil precursors, and basophils. This leads to reduced signaling and also an almost complete depletion of target cells, through antibody-mediated cytotoxicity.^23^ The CALIMA, SIROCCO, and ZONDA studies revealed its safety and efficacy in SA, with a 50% reduction in OCS dosage compared to placebo and a 70% reduction in annual exacerbations.^24^, ^25^, ^26^, ^27^ The phase III trial OSTRO showed that the addition of benralizumab every 8 weeks to the standard therapy of INCS (compared to placebo plus INCS), improved NPS and Nasal Blockage Score (NBS) at 40 weeks but did not improve SNOT-22 score or time to first nasal polyps surgery.^28^ In Italy, benralizumab is currently approved only for SEA.

Dupilumab is a completely human mAb that targets the alpha subunit of the interleukin 4 receptor (IL-4R), blocking both IL-4 and interleukin 13 (IL-13) signaling.^29^ A Phase III trial found that dupilumab, given subcutaneously once every 2 weeks, is more effective than placebo in reducing the frequency of asthma exacerbations and improving lung function and asthma control.^30^ The LIBERTY NP SINUS-24 and LIBERTY NP SINUS-54 studies showed that in individuals with CRSwNP dupilumab 300 mg every 2 weeks dramatically reduced the requirement for OCS compared to placebo, while also improving HRQoL measures using SNOT-22 and smell identification test.^4^ Currently, dupilumab in Italy is authorized both for SEA and CRSwNP.

SNOT-22 is a questionnaire constituted by 22 items that generates a total score that is a measure of HRQoL of patients suffering from CRS^31^ with or without NP, covering a series of symptoms that include physical problems, functional limitations, and emotional consequences.^32^ However, the SNOT-22 has expanded beyond its initial purpose of evaluating CRS therapy to become a widely used and clinically significant outcomes evaluation tool.^33^ SNOT-22 has become the standard questionnaire for measuring HRQoL of CRS in clinical trials;^4^^,^^18^^,^^22^^,^^28^ it has the advantage of considering both sinonasal and general health related symptoms^34^ The SNOT-22 has been divided into 4 key domains: nose symptoms, otologyc symptoms, sleep symptoms, and emotional symptoms.^31^

The purpose of this study is to examine the change in the overall SNOT-22 score and its relationship to the nasal, otologyc, sleep, and emotional domains in a real-life cohort of patients with CRSwNP during the first year of treatment with their first treatment with a biologic therapy drug at 1 (T1), 6 (T6), and 12 (T12) months.

## Methods

### Study design and patient population

In this prospective real-life observational study we enrolled 59 patients diagnosed with CRSwNP (in part having asthma as comorbidity) followed up by our allergology and clinical immunology unit. The period of enrollment started in May 2022 and ended in September 2022, with a median follow-up duration of 12 months. CRSwNP was diagnosed according to EPOS 2020 guidelines,^35^ through radiographic (maxillofacial computed tomography) or fibroscopy. The diagnosis of asthma was performed according to European Respiratory Society (ERS) guidelines.^35^ Patients with a past smoking history were included. We enrolled a total of 59 patients treated with mAb approved for CRSwNP and/or SA. For individuals with both CRSwNP and SA, we selected the mAb based on the information provided in the Summary of Product Characteristics (SPC) of each drug. For individuals with isolated CRSwNP, the drug used was dupilumab, as it was the first mAb approved for CRSwNP in Italy and the only one available for isolated CRSwNP until September 2022.

### Ethics, consent, and permissions

The study was performed according to the Helsinki Declaration, obtaining written consent from participants (SANI study approved on 23-07-2018; #15; MANI study approved on 05-10-2022 #5 by the AOU ethical committee). Each patients enrolled expressed written consent to participate to the study.

### Consent to publish

Each patient expressed written consent to publish and report individual patient data.

### Study design and measurements

Omalizumab was administered every 2 weeks or 4 weeks depending on total IgE at baseline and patient's weight; anti-IL5/5R was administered every 4 weeks or every 8 weeks according to the indication of the mAb; and anti-IL13/4R was administered every 4 weeks. We scheduled a visit at T0 (baseline), followed by a visit at T1 (1 month), T6 (6 months), and T12 (twelve months). At each visit, we administered the SNOT-22.

### Statistical Analysis

Comparisons of variables between groups were performed using the Mann–Whitney *U* test and the Kruskal–Wallis test, as appropriate. Statistical significance was established at p < 0.05. Data were analyzed using SPSS 23.0 for Windows (SPSS Inc., Chicago, IL, USA).

## Results

We enrolled 59 patients: 9 patients were treated with anti-IgE, 19 patients were treated with anti-IL5/5R and 30 patients were treated with anti-IL13/4R. The median age was 59 years (IQR 36.3–64.0) for patients treated with anti-IgE, 58.5 years (IQR 53.3–70.0) for patients treated with anti-IL-5/5R, and 53 years (IQR 42.7–59.0) for patients treated with anti-IL-13/4R (p = 0.01). All the patients treated with anti-IL5/5R had a diagnosis of asthma associated with CRSwNP, whereas in the groups treated with anti-IgE, 8 out of 9 patients had both asthma and CRSwNP, 1 was not asthmatic. In the group treated with anti-IL-13/4R, 22 out of 30 patients were asthmatic. In the anti-IgE group of patients, 6 were males and 3 were females; in the anti-IL5/5R, group 8 were males and 12 were females; and in the anti-IL-13/4R group, 10 were males and 20 were females (Table 1). At baseline the 3 groups were comparable in terms of body mass index (BMI), smoking history, and OCS use. There were no significant differences for non-biologic therapy of CRSwNP and asthma.

**Table 1:** Epidemiologic data of our cohort of patients

Epidemiological Data
**Gender (n of patients)**
Male 22
Female 37
**Age (years)**
Median 56
Max 21 – Min 76
**Comorbidities (% of patients)**
84.7%
66.1%
**Previous corticosteroid treatment (%of treatment)**
Nasal 100%
Systemic 100%
**Previous surgery ≥ 1 (% of patients)**
45.7%

In patients treated with anti-IgE mAb, a significant reduction in total score (p = 0.02) was observed between T0 and T1, but there was a non-significant reduction between T0 and T6 (p = 0.058) and between T0 and T12 (p = 0.09). There was not a significant reduction between T1 and T6 (p = 0.41), between T1 and T12 (p = 0.7), and between T6 and T12 (p = 0.1). (Table 2).

**Table 2:** Median value of SNOT-22 total score at each time point compared with the baseline, Median NPS value at 12 months compared with baseline.

mAb	Median SNOT-22 T0	Median SNOT-22 T1	Median SNOT-22 T6	Median SNOT-22 T12	SNOT-22 pT0 –T1	SNOT-22 pT0 –T6	SNOT-22 pT0 –T12	Median NPS value T0 – T12
Anti IgE	74 (IQR 52.2–82.3)	58 (IQR 29.0–73.5)	41 (IQR 8.3–66.0)	37 (IQR 26.8–66.0)	p = 0.025	p = 0.058	p = 0.09	7 (IQR 5.0–7.3) – 3 (IQR 0.7–3.7) (p = 0.0039)
Anti IL-5/5R	42 (IQR 36.5–63.8)	32 (IQR 24.5–39.8)	29 (IQR 23.5–37.7)	34 (IQR 16.3–37.8)	p = 0.001	p = 0.007	p = 0.052	
Anti IL-13/4R	63 (IQR 50.2–78.2)	25 (IQR 14.0–49.8)	15 (IQR 9.8–48.7)	18 (IQR 6.3–36.7)	p<0.0001	p<0.0001	p = 0.0005	

mAb: monoclonal antibody, NPS: Nasal Polyp Score; IQR: Interquartile range; SNOT-22: Sino-Nasal Outcome Test.

Considering those patients treated with anti-IL5/5R, we observed that the reduction in the SNOT-22 total score between T0 and T1 was significant (p = 0.001) as well as the reduction between T0 and T6 (p = 0.007), whereas between T0 and T12 the reduction was not significant (p = 0.052), and similarly the reduction between T1 and T6 (p = 0.59) was not statistically significant. Between T1 and T12 and between T6 and T12 there was an increase in SNOT-22 total score that was not statistically significant (p = 1.00 and p = 0.52 respectively). (Table 2).

In patients treated with anti-IL13/4R the difference in the SNOT-22 between T0 and T1 was significant (p < 0.0001), and similarly when comparing T0 and T6 (p < 0.0001) and also T0 and T12 (p = 0.0005) (Fig. 3). The difference between T1 and T6 was not significant (p = 0.07), but there was a further significant score reduction between T1 and T12 (p = 0.034); between T6 a T12 there was little but significant increase in SNOT-22 total score (p = 0.0067) (Table 2).

**Fig. 1:**
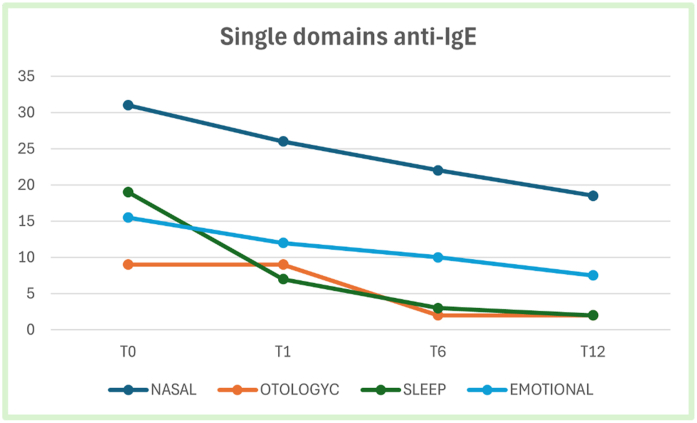
SNOT-22 single domains score median value at each timepoint in patients treated with anti-IgE

Analyzing the variation over time of individual domains of SNOT-22 in patients treated with anti-IgE we observed a significant reduction only in emotional domain between T0 and T12 (p = 0.03), while no significant reduction was found between T0 and T1 (p = 0.56) or T0 and T6 (p = 0.1). No significant reduction was found in nasal domain between T0 and T1 (p = 0.31) and T0 and T6 (p = 0.41) or T0 and T12(p = 0.15). Considering the otologyc domain, no significant reduction was found between T0 and T1 (p = 0.56) and T0 and T6 (p = 0.1) or T0 and T12 (p = 1). Finally for the sleep domain, no significant reduction was found neither between T0 and T1 (p = 0.08) and T0 and T6 (p = 0.25) or T0 and T12 (p = 0.47). (Fig. 1).

**Fig. 2:**
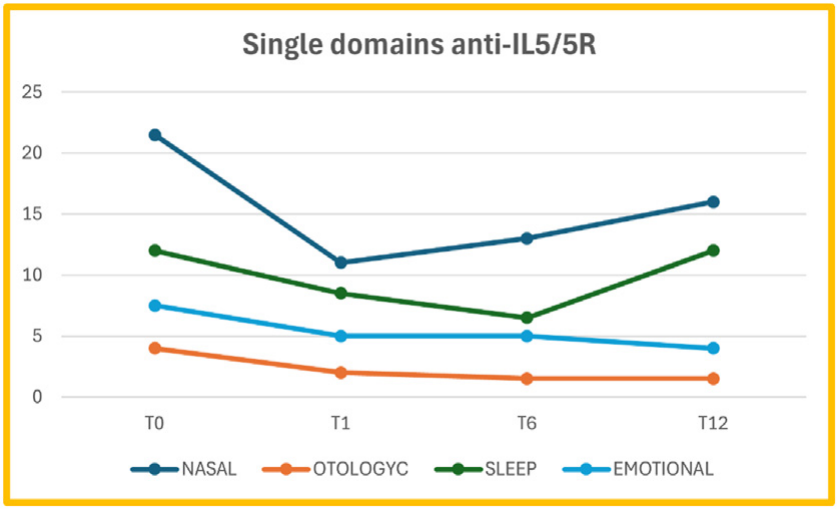
SNOT-22 single domains score median value at each timepoint in patients treated with anti-IL5/5R

In patients treated with anti-IL5/5R mAbs there was a significant difference in the nasal domain between T0 and T1 (p = 0.0009), T0 and T6 (p = 0.007), and between T0 and T12 (p = 0.04); for the otologyc domain, there was a significant difference between T0 and T1 (p = 0.003), between T0 and T6 (p = 0.03), but not between T0 and T12 (p = 0.19). For the sleep domain there was not a significant reduction between T0 and T1 (p = 0.76), neither between T0 and T6 (p = 0.36) or between T0 and T12(p = 0.06), while for the emotional domain there was a significant reduction between T0 and T12 (p = 0.01), but not between T0 and T1 (p = 0.36) or between T0 and T6 (p = 0.31) (Fig. 2).

**Fig. 3:**
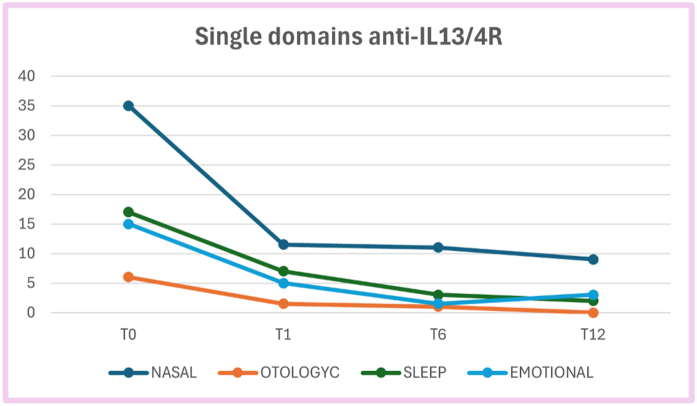
SNOT-22 single domains score median value at each timepoint in patients treated with anti-IL13/4R

In those treated with anti-IL13/4R mAb there was a significant reduction in the nasal domain between T0 and T1 (p < 0.0001), between T0 and T6 (p < 0.0001), and between T0 and T12 (p = 0.0009); for the otologyc domain, there was a significant reduction between T0 and T1 (p = 0.0001), between T0 and T6 (p = 0.002), and between T0 and T12 (p = 0.03); for the sleep domain, there was a significant reduction between T0 and T1 (p < 0.0001), between T0 and T6 (p < 0.0001), and between T0 and T12 (p = 0.0009). For the emotional domain there was a significant reduction between T0 and T1 (p < 0.0001), between T0 and T6 (p < 0.0001), and between T0 and T12 (p = 0.007). (Fig. 3). We conducted a sub analysis of SNOT-22 kinetics over time based on the positivity for skin prick test (SPT) for inhalant allergens. SPT had been previously performed in patients who had a clinical history suggestive for allergic rhinosinusitis; n = 31 (52,5%) showed a positivity for SPT, with Dermatophagoides pteronyssinus and Dermatophagoides farinae as the most common allergens. Considering patients with positive SPT we found a significant reduction of SNOT-22 between T0 and T6 (p < 0.0001) and between T0 and T12 (p = 0.003). Similarly, in patients with negative SPT we found a significant reduction between T0 and T6 (p < 0.0001) and between T0 and T12 (p = 0.004).

In patients affected by N-ERD we found a significant reduction in SNOT-22 between T0 and T6 (p = 0.003) but not between T0 and T12 (p = 0.08). Considering patients without N-ERD, we found a significant difference between T0 and T6 (p < 0.0001) and between T0 and T12 (p = 0.0002).

During the study period there was no Severe Adverse Event (SAE), in agreement with the previously reported good safety profile of mAb for SA and/or CRSwNP.^36^ SAE is intended as any undesirable event associated with the treatment whose outcome was death or life threatening, hospitalization (initial or prolonged), cause disability or permanent damage or requiring intervention to prevent disability or permanent damage. The number of adverse events (AEs) were comparable between groups, the most common was local reaction around site of injection. No patient interrupted the treatment due to AEs.

## Discussion

In this real-life prospective observational study, we found a significant SNOT-22 total score reduction for patients treated with anti-IgE after 1 month, but this significant difference was not maintained at 6 or 12 months compared with the baseline. The use of anti-IL5 led to a significant reduction of the SNOT-22 total score after 1 month, which was maintained at 6 months but not at 12 months. The use of an anti-IL13/4R resulted in a statistically significant reduction of the SNOT-22 score after 1 month of therapy, which was maintained later on at 6 and 12 months. Furthermore, only patients treated with anti-IL13/4R mAb showed a further significant improvement between 1 month and 12 months, highlighting that a significant response occurs already after 1 month but a further improvement may be subsequently expected during the first year of treatment.

In our cohort, much of the reduction in the SNOT-22 score for every mAb occurred within the first months of treatment. In the randomized placebo-controlled trials POLYP 1 and POLYP 2 it has been shown that an improvement in SNOT-22 score in patient treated with omalizumab is found at 4 weeks,^18^ consistently with our results. However, in these studies the SNOT-22 declines significantly at 24 weeks, while we did not find a significant difference at 6 or 12 months compared with the baseline. In a real-life study, Maza-Solano et al compared patients treated with ESS plus omalizumab with patients treated with either omalizumab or ESS alone and with a control group at 2 years. The omalizumab alone arm, which was comparable with our cohort for SNOT-22 at baseline (n = 9 patients, median SNOT-22 at baseline of 78, IQR 24.5) but with a lower mean age (mean 51.8 SD 10.5), showed a significant improvement in SNOT-22 score only at 12 months.^37^ A real-life study on 22 patients affected by CRSwNP treated with omalizumab for 24 weeks, showed a significant reduction in SNOT-22 score at 16 and 24 weeks,^38^ but this cohort was of younger age and had a lower SNOT-22 at baseline. In another real-life study on a population of 13 patients that was comparable to our cohort for age, the SNOT-22 score at 16 weeks showed a reduction of about 1.34-fold in mean value compared with the baseline (p < 0.0001).^39^

The clinical trial SYNAPSE^22^ showed that mepolizumab significantly reduced the SNOT-22 total score compared with placebo after 52 weeks of treatment, while the trial OSTRO showed similar results for benralizumab in CRSwNP at 52 weeks.^28^ In a real-life multicentric study, Gallo et al found a significant reduction in SNOT-22 total score at 52 weeks of treatment with mepolizumab compared with the baseline, but in this cohort the mean baseline SNOT-22 (mean 54.8 ± 25.9) was lower that in our cohort.^40^ Another real-life study of Domínguez-Sosa et al showed the effectiveness of mepolizumab on SNOT-22 total score at 24 weeks (delta SNOT-22: 63 points; p < 0.001) in a cohort of 55 patients, that is comparable with our results.^41^ Few real-life studies are present in literature about the effectiveness of benralizumab in CRSwNP, particularly those assessing the SNOT-22 and its evolution beyond 6 months of treatment. Santomasi et al. found a significant reduction in SNOT-22 at 52 weeks of treatment with benralizumab in a cohort of 17 patients comparable with our cohort for age and number of patients.^42^ In our study we found a significant reduction in SNOT-22 total score in the group of patients treated with mepolizumab or benralizumab at T1 and T6 compared to the baseline, but not at T12. We have to consider that at baseline the median SNOT-22 of the IL-5/5R group was lower compared with that of the other 2 groups (Table 2).

Our results for dupilumab are comparable with both randomized clinical trials as LIBERTY NP SINUS-24 and LIBERTY NP SINUS-52^4^ and real-life studies as DUPIREAL,^42^ where the SNOT-22 scores decrease significantly (p < 0.001) from baseline to 9 months and then reached stable values, in a cohort of patients greater than our (n = 648) but comparable for age (median 54, IQR 45–63), and SNOT-22 at baseline (median score 58.0, IQR 49.0–70.0).^43^

Another real-life study on dupilumab in a cohort of 81 patients comparable with our cohort for age (mean 49.72 ± 14.2), showed a significant reduction of SNOT-22 at 3, 6, and 12 months (p = 0.0001, p = 0.005, p = 0.005), comparable with our results.^44^

The real-life studies available in literature for omalizumab, mepolizumab, and benralizumab were often inconsistent with our results, but they evaluated cohorts that were younger and/or with a lower baseline SNOT-22 and/or had a shorter follow up (16–24 weeks). Furthermore, our data are consistent with real-life studies on dupilumab that consider cohorts of patients close to ours for age, SNOT-22 at baseline and time of follow-up. In our opinion, given these inconsistencies, more studies are needed to assess the effectiveness of these biologic in CRSwNP in real-life, according to the complexity and variability of patients with respect to registrative trials.

When examining the single domains, we observed that patients who received anti-IL13/4R treatment demonstrated an improvement in each domain at each time point (T) compared to the baseline. Patients who received anti-IL5/5R treatment demonstrated an improvement in the nasal domain at each T compared to the baseline. However, the improvement in the otologyc domain was not sustained after 12 months. Similarly, the sleep domain remained unchanged, and the emotional domain only improved after 12 months relative to the baseline. Similarly, there was a reduction of the emotional domain in patients treated with anti-IgE; furthermore, it was the only domain in which these patients demonstrated a significant improvement also at 12 months.

CRSwNP is a chronic and recurrent condition, requiring long-term management. While treatment options such as INCS, SCS and ESS can often alleviate symptoms and improve HRQoL, there are possible short- and long-term side effects and risks and NP may recur even after successful surgery. Patients may experience frustration with the management of their disease and perceive that the impact on their HRQoL is not adequately acknowledged. The implementation of specific biologicals in the treatment armamentarium should result ideally in a stable improvement in HRQoL for patients affected by CRSwNP.^45^

Dupilumab was the first drug approved in Italy for SEA and CRSwNP, followed by omalizumab and mepolizumab, while benralizumab is currently only approved for the SEA. Patients of our cohort were either patients with SEA and CRSwNP as comorbidity treated with the said mAbs for this clinical indication or patients with CRSwNP that fitted the criteria for starting treatment with mAbs. The focus of medical treatment of CRSwNP is primarily to treat inflammation to reduce polyps’ size and improve symptoms and HRQoL.^46^ In the biologics era, a further goal may be the remission of the disease.^47^ Various consensuses and studies very recently discussed the concept of remission in asthma, reaching to share a definition of clinical remission. In some countries such as Germany, Spain, and Japan, the national guidelines included these definitions for SA.^48^ For CRSwNP a consensus on the definition of remission is currently debated but in the European Position Paper On Rhinosinusitis And Nasal Polyps (EPOS2020) the concept of disease control was defined based on nasal endoscopy results and need of rescue treatment in the last 6 months, but a particular emphasis was given to symptoms improvement. In particular the nasal blockage, rhinorrhea/postnasal drip, facial pain/pressure, smell, sleep disturbance or fatigue are items captured by SNOT-22 questionnaire.^35^ In a recent EPOS2020/EUFOREA expert opinion, the remission was defined by the presence of sustained control for almost 12 months combined with the absence of active disease evaluated by nasal endoscopy.^49^ Furthermore, a recent paper by Caminati et al offers a suggestion about a possible definition of remission: the author proposed a series of objective and subjective criteria to define the remission in CRSwNP.^47^ Among these criteria an important role is given to nasal symptoms like nasal obstruction, loss of smell, rhinorrhea, and craniofacial pain and no impact of symptoms recurrence as evaluated by SNOT-22. According to a recent study conducted in a real-life setting, patients who had worse nasal symptoms, a reduced sense of smell, and high scores on computed tomography using the Lund-Mackay score had greater risk of experiencing RNPs. This risk was also observed in patients with allergies and higher levels of blood and tissue eosinophils. However, the relative prognostic significance of these risk factors has not been investigated.^50^

From the aforementioned studies we can hypothesize that the role of symptom resolution and patient HRQoL will be pivotal in the definition of disease remission. Therefore, analyzing the effectiveness of different mAbs in reducing symptoms and improving HRQoL measured by SNOT-22 questionnaire, will be crucial in particular on the medium and long-term.

To date, to our knowledge there are no published data of the change in SNOT-22 single domains before and after biologic treatment for asthma or CRSwNP. Förster-Ruhrmann et al in their study consider the HRQoL in patients affected by CRSwNP treated with type 2 (T2) mAb before and after 4–6 months of treatment, but they used the Rhinosinusitis Outcome Measure 31 (RSOM-31), and its subscale scores (nose, eye, ear, general, practical problems, emotion) and the evaluation of nasal symptoms such blocked nose, loss of smell, runny nose, postnasal drip using the Visual Analogue Scale (VAS). In this study, the authors found a better response to dupilumab in rhinologic parameters, close to our results, although RSOM-31 total score reduced significantly compared with the baseline, independently of the mAb considered.^51^ The observation that in our cohort those patients treated with both IL5/5R and IL13/4R experienced an improvement in the nasal domain demonstrates the effectiveness of these 2 mAbs against nasal symptoms. It is crucial to note that 68.6% of patients of our cohort reached a reduction in SNOT-22 total score better than the MCID of 8.9 points already after 1 month of therapy,^21^ independently from the type of mAb. This percentage improved at 12 months (70.6%). This may confirm that a good response was achieved already after 1 month of therapy and may account for the improvement in the emotional domain observed in every mAb, which may be indicative of the overall improvement observed in this cohort of patients irrespectively from the mAb. In parallel with this, the median NPS of our cohort decreased significantly from a median of 7 (IQR 5–7.3) to 3 (IQR 0.7–3.7) (p = 0.0039) after 12 months of therapy. (Table 2).

A systematic review and network meta-analysis of 3400 patients affected by CRSwNP, treated with omalizumab, mepolizumab, benralizumab, and dupilumab compared to the standard of care showed a moderate to high certainty of evidence in improving the HRQoL. In particular, the authors found that omalizumab, mepolizumab, and dupilumab all resulted in a reduction in the mean score of the SNOT-22 that was greater than the MCID. This is comparable to our findings. In addition, in this meta-analysis, patients who were treated with omalizumab and dupilumab showed the greatest reduction, which is comparable to the results that we obtained.^52^(Table 2). Furthermore, the same study found a moderate to high certainty of efficacy of omalizumab, mepolizumab, benralizumab, and dupilumab in reducing NPS.

Our study had several limitations including the small number of patients, the non-randomized study design, the reduced ethnic diversity, and the non-homogenous population in terms of asthma comorbidity or severity. However, the time of follow-up is longer than available published real-life studies and importantly we consider single domains of SNOT-22.

Our study provides a comprehensive analysis of the kinetics throughout the first year of treatment with mAbs in patients with CRSwNP. The results show distinct patterns in symptom reduction and improvement in HRQoL. The longitudinal evaluation of SNOT-22 can aid in promptly distinguishing patients who respond quickly to mAbs from those who have a low or no response. It will probably can also help in the near future in accurately defining clinical remission induced by targeted treatments.

## Abbreviations

CRSwNP: Chronic Rhinosinusitis with Nasal Polyps; SEA: Severe Eosinophilic Asthma; SA: Severe Asthma; N-ERD: Non-Steroidal Anti-Inflammatory Drugs-exacerbated respiratory disease; SNOT-22: Sino-Nasal Outcome Test 22; mAb: monocolonal Antibodies; ACT: Asthma Control Test; HRQoL: Health Related Quality Of Life; NPS: Nasal Polyp Score; MCID: Minimal Clinically Important Difference; SPC: Summary of Product Characteristics; SPT: Skin Prick Test; RNPs: Recurrent Nasal Polyps; IL-5: Interleukin 5; IL-5R: Interleukin 5 receptor; IL-4: Interleukin 4; IL-13: Interleukin 13; VAS: Visual Analogue Scale; RSOM31: Rhinosinusitis Outcome Measure-31.

## Consent to participate

Written informed consent is recorded for each patient.

## Availability of data and materials

Data will be made available upon reasonable request from the corresponding author, GAML. Costanzo.

## Author contributions

Conceptualization: Giulia Anna Maria Luigia Costanzo, Andrea Giovanni Ledda, Stefano Del Giacco and Davide Firinu; Methodology: Giulia Anna Maria Luigia Costanzo, Andrea Giovanni Ledda, Giada Sambugaro Stefano Del Giacco and Davide Firinu; Formal analysis Stefano Del Giacco and Davide Firinu; Investigation: Giulia Anna Maria Costanzo, Andrea Giovanni Ledda, Giada Sambugaro and Silvia Canalis; Data curation: Giulia Anna Maria Costanzo, Andrea Giovanni Ledda, Giada Sambugaro, Silvia Canalis, Maria Pina Barca, Stefano Del Giacco and Davide Firinu; Writing—original draft preparation: Giulia Anna Maria Costanzo, Andrea Giovanni Ledda, Stefano Del Giacco and Davide Firinu; Writing review and editing: Giulia Anna Maria Luigia Costanzo, Andrea Giovanni Ledda, Giuseppe Murdaca, Cristiano Caruso, Stefano Del Giacco and Davide Firinu; Supervision: Stefano Del Giacco and Davide Firinu; Project administration: Stefano Del Giacco and Davide Firinu.

## Ethics approval

SANI study approved on 23-07-2018; #15; MANI study approved on 05-10-2022 #5 by the AOU ethical committee.

## Authors' consent for publication

All authors have read and agreed to the published version of the manuscript.

## Consent for publication

Written informed consent is recorded for each patient.

## Funding

This research received no external funding.

## Declaration of competing interest

GAML. Costanzo declares no competing interests,

AG. Ledda declares no competing interests,

G. Sambugaro declares no competing interests,

G. Murdaca declares no competing interests,

C. Caruso declares no competing interests,

S. Canalis declares no competing interests,

P. Serra declares no competing interests,

MP. Barca declares no competing interests,

S. Del Giacco has received speaker fees by AstraZeneca, Chiesi, GSK, Guidotti, Menarini, Novartis, Sanofi, Stallergenes, Takeda and Valeas. He has received advisory board fees from AstraZeneca, Chiesi, CSL-Behring, GSK, Novartis, Sanofi and Takeda, and has received research grants from AstraZeneca, CSL.Behring, GSK and Novartis.

D. Firinu declare no competing interests.
